# Bis(μ-2-phenyl­quinoline-4-carboxyl­ato)bis­[aqua­(1,10-phenanthroline)(2-phenyl­quinoline-4-carboxyl­ato)manganese(II)] dihydrate

**DOI:** 10.1107/S1600536811039341

**Published:** 2011-09-30

**Authors:** Wei-Wei Li, Yue Bing, Mei-Qin Zha, Tian-Hua Li, Xing Li

**Affiliations:** a Faculty of Materials Science and Chemical Engineering, Ningbo University, Ningbo 315211, People’s Republic of China

## Abstract

In the centrosymmetric dinuclear title complex, [Mn_2_(C_16_H_10_NO_2_)_4_(C_12_H_8_N_2_)_2_(H_2_O)_2_]·2H_2_O, the Mn^II^ cation is in a distorted octa­hedral coordination geometry defined by two N atoms from a 1,10-phenanthroline ligand, one water O atom and three O atoms from three 2-phenyl­quinoline-4-carboxyl­ate anions. A pair of 2-phenyl­quinoline-4-carboxyl­ate anions bridge two Mn cations, forming the dinuclear mol­ecule. An intra­moleculr O—H⋯O hydrogen bond occurs. Inter­molecular O—H⋯O and O—H⋯N hydrogen bonds are present in the crystal structure.

## Related literature

For applications of coordination polymers, see: Wang *et al.* (2009 ▶); Xi *et al.* (2009 ▶); Xu *et al.* (2008 ▶); Ferey (2008 ▶). For a related structure, see: Shen *et al.* (2007 ▶).
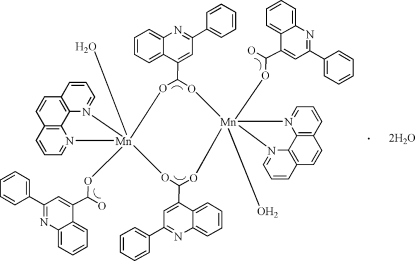


## Experimental

### 

#### Crystal data

[Mn_2_(C_16_H_10_NO_2_)_4_(C_12_H_8_N_2_)_2_(H_2_O)_2_]·2H_2_O
*M* *_r_* = 1535.35
Monoclinic, 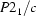

*a* = 14.926 (4) Å
*b* = 13.847 (4) Å
*c* = 17.717 (5) Å
β = 96.919 (4)°
*V* = 3635.1 (18) Å^3^
*Z* = 2
Mo *K*α radiation
μ = 0.42 mm^−1^
*T* = 296 K
0.35 × 0.15 × 0.12 mm

#### Data collection

Bruker SMART CCD area-detector diffractometer
Absorption correction: multi-scan (*SADABS*; Bruker, 2001 ▶) *T*_min_ = 0.927, *T*_max_ = 0.951
31242 measured reflections
8315 independent reflections
5656 reflections with *I* > 2σ(*I*)
*R*_int_ = 0.044

#### Refinement

*R*[*F* ^2^ > 2σ(*F* ^2^)] = 0.039
*wR*(*F* ^2^) = 0.113
*S* = 1.03
8315 reflections
512 parameters
4 restraints
H atoms treated by a mixture of independent and constrained refinement
Δρ_max_ = 0.41 e Å^−3^
Δρ_min_ = −0.28 e Å^−3^

### 

Data collection: *SMART* (Bruker, 2007 ▶); cell refinement: *SAINT* (Bruker, 2007 ▶); data reduction: *SAINT*; program(s) used to solve structure: *SHELXTL* (Sheldrick, 2008 ▶); program(s) used to refine structure: *SHELXTL*; molecular graphics: *SHELXTL*; software used to prepare material for publication: *SHELXTL*.

## Supplementary Material

Crystal structure: contains datablock(s) I, global. DOI: 10.1107/S1600536811039341/xu5330sup1.cif

Structure factors: contains datablock(s) I. DOI: 10.1107/S1600536811039341/xu5330Isup2.hkl

Additional supplementary materials:  crystallographic information; 3D view; checkCIF report

## Figures and Tables

**Table 1:** Selected bond lengths (Å)

Mn1—O1	2.1740 (14)
Mn1—O3	2.1557 (15)
Mn1—O4^i^	2.1148 (14)
Mn1—O5	2.2358 (16)
Mn1—N3	2.2706 (16)
Mn1—N4	2.2914 (15)

Symmetry code: (i) 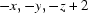.

**Table 2:** Hydrogen-bond geometry (Å, °)

*D*—H⋯*A*	*D*—H	H⋯*A*	*D*⋯*A*	*D*—H⋯*A*
O5—H5*A*⋯O2	0.85 (2)	1.81 (2)	2.630 (2)	161 (3)
O5—H5*B*⋯N2^ii^	0.83 (2)	2.04 (2)	2.868 (2)	176
O6—H6*A*⋯O1^iii^	0.89 (5)	2.30 (5)	3.175 (3)	167
O6—H6*B*⋯N1^iv^	0.87 (2)	2.23 (2)	3.051 (3)	157

Symmetry codes: (ii) 
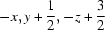
; (iii) 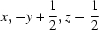; (iv) 
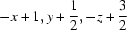
.

**Fig. 1.**
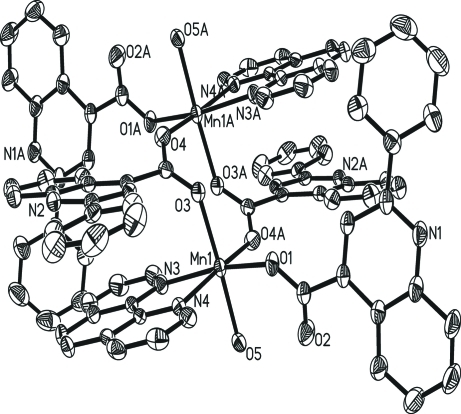
A view of the manganese ion coordination, showing the atoms 30% probability ellipsoids. Guest water molecules and H atoms have been omitted for clarity.

**Fig. 2.**
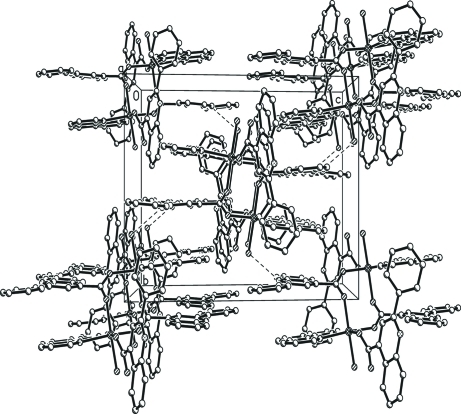
Packing of title complex showing the three dimensional hydrogen bonding network.

## supplementary crystallographic information

### Comment

Crystal engineering of coordination polymers have attracted a considerable ongoing research in the past few decades because of their variety of topological architectures and the diverse fascinating functionalities for potential applications (Ferey, 2008; Wang *et al.*, 2009). 2-phenylquinoline-4-carboxylic acid, aside from the significance in biological systems (Xi *et al.*, 2009; Xu *et al.*, 2008), also possesses fascinating coordination behaviors, such as asymmetric geometry and multiple coordination sites, which has been widely used to design and synthesize metal-organic coordination complexes because of the carboxylate group and/or pyridine nitrogen atom (Shen *et al.*, 2007). Herein we report the preparation and characterization of a new 2-phenylquinoline-4-carboxylate-manganese(II) complex, [Mn_2_(C_16_H_10_NO_2_)_4_(Phen)_2_(H_2_O)_2_].2H_2_O.

Single-crystal X-ray diffraction analysis indicates the title complex possesses a dinuclear structure. The asymmetric unit consists of four ligands, two phen ligands, two manganese ions, two coordinated water molecules and two guest water molecules. A view of the manganese ion coordination is shown in Figure 1, where the metal center is coordinated in an octahedral geometry by two N atoms from one phen with Mn—N distances ranging 2.2706 (16) and 2.2914 (15) Å and four O atoms from three ligands and one coordinated water molecule with Mn—O distances ranging from 2.1148 (14) to 2.2358 (16) Å. The intermolecular O—H···O and O—H···N hydrogen bondings is helpful to the stabilization of the crystal structure (Figure 2).

### Experimental

2-Phenylquinoline-4-carboxylic acid (0.0123 g, 0.05 mmol), Mn(OAc)_2_.2H_2_O (0.0250 g, 0.10 mmol), phen (0.0198 g, 0.10 mmol) and KOH (0.0028 g, 0.05 mmol) in H_2_O solution (10 ml) were placed in a 25 ml stainless reactor fitted with a Teflon liner and heated to 373 K for two days, then cooled to room temperature, yellow block like crystals were obtained (yield, 50%).

### Refinement

H atoms attached to C atoms were placed in calculated positions and treated using a riding-model approximation [C–H = 0.95–0.98 with *U*_iso_(H) = 1.2Ueq(C)/1.5Ueq(C)]. H atoms bonded to O atoms were visible in the difference Fourier map and refined freely.

### Crystal data

**Table:**

[Mn_2_(C_16_H_10_NO_2_)_4_(C_12_H_8_N_2_)_2_(H_2_O)_2_]·2H_2_O	*F*(000) = 1588
*M**_r_* = 1535.35	*D*_x_ = 1.403 Mg m^−^^3^
Monoclinic, *P*2_1_/*c*	Mo *K*α radiation, λ = 0.71073 Å
Hall symbol: -P 2ybc	Cell parameters from 6315 reflections
*a* = 14.926 (4) Å	θ = 2.2–25.3°
*b* = 13.847 (4) Å	µ = 0.42 mm^−^^1^
*c* = 17.717 (5) Å	*T* = 296 K
β = 96.919 (4)°	Block, light-yellow
*V* = 3635.1 (18) Å^3^	0.35 × 0.15 × 0.12 mm
*Z* = 2	

### Data collection

**Table:**

Bruker SMART CCD area-detector diffractometer	8315 independent reflections
Radiation source: fine-focus sealed tube	5656 reflections with *I* > 2σ(*I*)
graphite	*R*_int_ = 0.044
φ and ω scans	θ_max_ = 27.5°, θ_min_ = 1.9°
Absorption correction: multi-scan (*SADABS*; Bruker, 2001)	*h* = −17→19
*T*_min_ = 0.927, *T*_max_ = 0.951	*k* = −17→17
31242 measured reflections	*l* = −22→22

### Refinement

**Table:**

Refinement on *F*^2^	Primary atom site location: structure-invariant direct methods
Least-squares matrix: full	Secondary atom site location: difference Fourier map
*R*[*F*^2^ > 2σ(*F*^2^)] = 0.039	Hydrogen site location: inferred from neighbouring sites
*wR*(*F*^2^) = 0.113	H atoms treated by a mixture of independent and constrained refinement
*S* = 1.03	*w* = 1/[σ^2^(*F*_o_^2^) + (0.0558*P*)^2^ + 0.2051*P*] where *P* = (*F*_o_^2^ + 2*F*_c_^2^)/3
8315 reflections	(Δ/σ)_max_ = 0.001
512 parameters	Δρ_max_ = 0.41 e Å^−^^3^
4 restraints	Δρ_min_ = −0.28 e Å^−^^3^

### Special details

**Table:**

Geometry. All e.s.d.'s (except the e.s.d. in the dihedral angle between two l.s. planes) are estimated using the full covariance matrix. The cell e.s.d.'s are taken into account individually in the estimation of e.s.d.'s in distances, angles and torsion angles; correlations between e.s.d.'s in cell parameters are only used when they are defined by crystal symmetry. An approximate (isotropic) treatment of cell e.s.d.'s is used for estimating e.s.d.'s involving l.s. planes.
Refinement. Refinement of *F*^2^ against ALL reflections. The weighted *R*-factor *wR* and goodness of fit *S* are based on *F*^2^, conventional *R*-factors *R* are based on *F*, with *F* set to zero for negative *F*^2^. The threshold expression of *F*^2^ > σ(*F*^2^) is used only for calculating *R*-factors(gt) *etc*. and is not relevant to the choice of reflections for refinement. *R*-factors based on *F*^2^ are statistically about twice as large as those based on *F*, and *R*- factors based on ALL data will be even larger.

### Fractional atomic coordinates and isotropic or equivalent isotropic displacement parameters (Å^2^)

**Table:**

	*x*	*y*	*z*	*U*_iso_*/*U*_eq_	
Mn1	0.077680 (18)	0.13501 (2)	0.951424 (14)	0.03543 (10)	
O1	0.22272 (9)	0.12800 (10)	0.98381 (8)	0.0504 (4)	
O2	0.24413 (10)	0.27644 (12)	1.03173 (10)	0.0738 (5)	
O3	0.06564 (9)	−0.01826 (10)	0.93138 (7)	0.0443 (3)	
O4	−0.04776 (10)	−0.12260 (10)	0.93530 (7)	0.0529 (4)	
O5	0.07358 (10)	0.29333 (10)	0.97558 (8)	0.0445 (3)	
O6	0.26767 (16)	0.5645 (2)	0.39858 (18)	0.1290 (10)	
N1	0.53501 (11)	0.09953 (14)	1.12225 (10)	0.0557 (5)	
N2	−0.03456 (11)	−0.08570 (11)	0.65569 (8)	0.0402 (4)	
N3	−0.06204 (10)	0.15689 (12)	0.88748 (9)	0.0425 (4)	
N4	0.09674 (10)	0.16239 (11)	0.82681 (8)	0.0373 (4)	
C1	0.27011 (13)	0.19290 (16)	1.01920 (11)	0.0463 (5)	
C2	0.36515 (12)	0.16419 (15)	1.05267 (10)	0.0428 (5)	
C3	0.38370 (13)	0.06909 (15)	1.06611 (10)	0.0447 (5)	
H3	0.3395	0.0234	1.0511	0.054*	
C4	0.43546 (13)	0.23362 (16)	1.07405 (11)	0.0488 (5)	
C5	0.42841 (15)	0.33411 (18)	1.06345 (15)	0.0666 (7)	
H5	0.3738	0.3605	1.0423	0.080*	
C6	0.50050 (18)	0.3939 (2)	1.08366 (18)	0.0839 (9)	
H6	0.4943	0.4602	1.0766	0.101*	
C7	0.58388 (18)	0.3551 (2)	1.11501 (19)	0.0895 (10)	
H7	0.6329	0.3958	1.1277	0.107*	
C8	0.59324 (16)	0.2594 (2)	1.12680 (16)	0.0788 (8)	
H8	0.6487	0.2349	1.1478	0.095*	
C9	0.51972 (13)	0.19506 (17)	1.10771 (12)	0.0555 (6)	
C10	0.46890 (13)	0.03769 (16)	1.10258 (11)	0.0479 (5)	
C11	0.48590 (14)	−0.06496 (17)	1.12264 (13)	0.0535 (5)	
C12	0.53980 (19)	−0.0895 (2)	1.18959 (17)	0.0868 (9)	
H12	0.5677	−0.0413	1.2206	0.104*	
C13	0.5518 (2)	−0.1856 (3)	1.2101 (2)	0.1048 (11)	
H13	0.5868	−0.2012	1.2554	0.126*	
C14	0.44751 (15)	−0.13894 (17)	1.07692 (15)	0.0610 (6)	
H14	0.4117	−0.1242	1.0318	0.073*	
C15	0.46197 (18)	−0.2346 (2)	1.09785 (18)	0.0808 (8)	
H15	0.4367	−0.2835	1.0662	0.097*	
C16	0.5133 (2)	−0.2577 (2)	1.1650 (2)	0.1006 (11)	
H16	0.5217	−0.3219	1.1795	0.121*	
C17	0.00152 (13)	−0.06932 (13)	0.90100 (10)	0.0393 (4)	
C18	−0.01582 (13)	−0.07117 (12)	0.81476 (9)	0.0361 (4)	
C19	0.05738 (13)	−0.06910 (12)	0.77495 (10)	0.0369 (4)	
H19	0.1150	−0.0627	0.8010	0.044*	
C20	−0.10419 (13)	−0.07755 (13)	0.77399 (10)	0.0378 (4)	
C21	−0.18619 (14)	−0.07534 (15)	0.80722 (12)	0.0502 (5)	
H21	−0.1846	−0.0707	0.8597	0.060*	
C22	−0.26733 (15)	−0.07998 (17)	0.76262 (13)	0.0592 (6)	
H22	−0.3204	−0.0778	0.7851	0.071*	
C23	−0.27174 (15)	−0.08801 (17)	0.68346 (13)	0.0588 (6)	
H23	−0.3275	−0.0922	0.6540	0.071*	
C24	−0.19513 (14)	−0.08966 (15)	0.64978 (12)	0.0511 (5)	
H24	−0.1989	−0.0945	0.5971	0.061*	
C25	−0.10912 (13)	−0.08409 (13)	0.69350 (10)	0.0388 (4)	
C26	0.04644 (13)	−0.07653 (12)	0.69469 (10)	0.0364 (4)	
C27	0.12600 (14)	−0.07413 (13)	0.65102 (11)	0.0427 (5)	
C28	0.11378 (16)	−0.06426 (16)	0.57216 (12)	0.0607 (6)	
H28	0.0557	−0.0602	0.5466	0.073*	
C29	0.1872 (2)	−0.0604 (2)	0.53144 (15)	0.0793 (8)	
H29	0.1778	−0.0548	0.4788	0.095*	
C30	0.21377 (15)	−0.07807 (16)	0.68732 (13)	0.0579 (6)	
H30	0.2236	−0.0844	0.7399	0.069*	
C31	0.28697 (18)	−0.0727 (2)	0.64598 (16)	0.0776 (8)	
H31	0.3454	−0.0746	0.6711	0.093*	
C32	0.2735 (2)	−0.0646 (2)	0.56783 (17)	0.0811 (8)	
H32	0.3225	−0.0620	0.5401	0.097*	
C33	−0.13864 (14)	0.15679 (16)	0.91749 (14)	0.0568 (6)	
H33	−0.1366	0.1491	0.9698	0.068*	
C34	−0.22241 (15)	0.16763 (18)	0.87456 (17)	0.0708 (7)	
H34	−0.2750	0.1682	0.8979	0.085*	
C35	−0.22613 (15)	0.17737 (18)	0.79814 (17)	0.0706 (7)	
H35	−0.2818	0.1837	0.7688	0.085*	
C36	−0.14736 (14)	0.17799 (15)	0.76307 (13)	0.0533 (6)	
C37	−0.06530 (12)	0.16867 (13)	0.81122 (11)	0.0394 (4)	
C38	−0.14444 (18)	0.18754 (17)	0.68263 (14)	0.0675 (7)	
H38	−0.1983	0.1933	0.6505	0.081*	
C39	−0.0665 (2)	0.18842 (16)	0.65274 (12)	0.0643 (7)	
H39	−0.0672	0.1953	0.6004	0.077*	
C40	0.01816 (15)	0.17908 (14)	0.69947 (10)	0.0460 (5)	
C41	0.01878 (13)	0.17047 (12)	0.77883 (10)	0.0364 (4)	
C42	0.10130 (18)	0.18032 (16)	0.67071 (12)	0.0590 (6)	
H42	0.1036	0.1870	0.6187	0.071*	
C43	0.17865 (17)	0.17165 (16)	0.71898 (12)	0.0579 (6)	
H43	0.2343	0.1713	0.7004	0.070*	
C44	0.17374 (14)	0.16325 (15)	0.79703 (11)	0.0462 (5)	
H44	0.2273	0.1580	0.8296	0.055*	
H5A	0.1296 (12)	0.3012 (19)	0.9910 (15)	0.092 (10)*	
H6A	0.246 (4)	0.511 (3)	0.418 (3)	0.27 (3)*	
H5B	0.0596 (16)	0.3279 (16)	0.9376 (11)	0.074 (8)*	
H6B	0.3245 (14)	0.556 (3)	0.393 (2)	0.147 (16)*	

### Atomic displacement parameters (Å^2^)

**Table:**

	*U*^11^	*U*^22^	*U*^33^	*U*^12^	*U*^13^	*U*^23^
Mn1	0.03206 (16)	0.04590 (18)	0.02736 (14)	−0.00132 (13)	−0.00034 (10)	0.00228 (12)
O1	0.0319 (7)	0.0636 (10)	0.0529 (8)	0.0000 (7)	−0.0070 (6)	−0.0057 (7)
O2	0.0458 (9)	0.0658 (11)	0.1038 (13)	0.0064 (8)	−0.0157 (9)	−0.0141 (10)
O3	0.0499 (8)	0.0459 (8)	0.0357 (7)	−0.0008 (6)	−0.0006 (6)	−0.0022 (6)
O4	0.0740 (10)	0.0543 (9)	0.0314 (7)	−0.0159 (7)	0.0106 (7)	0.0016 (6)
O5	0.0489 (9)	0.0483 (9)	0.0351 (7)	0.0036 (7)	0.0004 (6)	0.0055 (6)
O6	0.0584 (15)	0.160 (3)	0.165 (3)	−0.0228 (16)	−0.0017 (15)	0.058 (2)
N1	0.0352 (10)	0.0640 (12)	0.0643 (11)	0.0023 (9)	−0.0086 (8)	−0.0085 (10)
N2	0.0482 (10)	0.0409 (9)	0.0310 (8)	−0.0013 (7)	0.0028 (7)	−0.0004 (7)
N3	0.0327 (9)	0.0501 (10)	0.0436 (9)	0.0020 (7)	−0.0003 (7)	0.0045 (7)
N4	0.0392 (9)	0.0388 (9)	0.0333 (8)	−0.0007 (7)	0.0021 (7)	0.0023 (6)
C1	0.0342 (11)	0.0598 (14)	0.0438 (11)	0.0004 (10)	−0.0007 (9)	−0.0015 (10)
C2	0.0312 (10)	0.0594 (13)	0.0370 (10)	−0.0011 (9)	0.0013 (8)	−0.0040 (9)
C3	0.0324 (10)	0.0601 (14)	0.0411 (10)	−0.0051 (9)	0.0020 (8)	−0.0039 (9)
C4	0.0360 (11)	0.0587 (14)	0.0507 (11)	−0.0005 (10)	0.0005 (9)	−0.0075 (10)
C5	0.0440 (13)	0.0629 (16)	0.0895 (18)	−0.0001 (11)	−0.0059 (12)	−0.0023 (13)
C6	0.0644 (18)	0.0606 (16)	0.123 (2)	−0.0089 (14)	−0.0045 (16)	−0.0095 (16)
C7	0.0492 (16)	0.076 (2)	0.136 (3)	−0.0146 (14)	−0.0169 (16)	−0.0197 (18)
C8	0.0416 (13)	0.0723 (18)	0.115 (2)	−0.0048 (12)	−0.0214 (13)	−0.0172 (16)
C9	0.0342 (11)	0.0675 (16)	0.0622 (13)	−0.0025 (10)	−0.0045 (10)	−0.0117 (11)
C10	0.0325 (11)	0.0664 (15)	0.0438 (11)	0.0006 (10)	0.0002 (8)	−0.0043 (10)
C11	0.0356 (11)	0.0649 (15)	0.0607 (13)	0.0041 (10)	0.0086 (10)	0.0064 (11)
C12	0.0719 (19)	0.091 (2)	0.089 (2)	0.0023 (16)	−0.0221 (15)	0.0191 (17)
C13	0.081 (2)	0.107 (3)	0.120 (3)	0.008 (2)	−0.0144 (19)	0.048 (2)
C14	0.0473 (13)	0.0657 (16)	0.0718 (15)	0.0024 (11)	0.0153 (11)	−0.0016 (13)
C15	0.0659 (17)	0.0637 (18)	0.115 (2)	−0.0006 (14)	0.0207 (16)	0.0011 (16)
C16	0.070 (2)	0.074 (2)	0.158 (3)	0.0129 (17)	0.017 (2)	0.034 (2)
C17	0.0510 (12)	0.0358 (10)	0.0318 (9)	0.0030 (9)	0.0074 (8)	−0.0016 (8)
C18	0.0508 (12)	0.0271 (9)	0.0304 (9)	−0.0017 (8)	0.0050 (8)	−0.0009 (7)
C19	0.0452 (11)	0.0322 (10)	0.0329 (9)	−0.0028 (8)	0.0027 (8)	−0.0007 (7)
C20	0.0457 (11)	0.0300 (10)	0.0383 (10)	0.0012 (8)	0.0079 (8)	0.0007 (8)
C21	0.0544 (13)	0.0543 (13)	0.0432 (11)	0.0020 (10)	0.0108 (10)	−0.0009 (9)
C22	0.0465 (13)	0.0728 (16)	0.0600 (14)	0.0053 (11)	0.0131 (11)	−0.0003 (12)
C23	0.0471 (13)	0.0681 (15)	0.0593 (14)	0.0024 (11)	−0.0012 (11)	0.0014 (12)
C24	0.0522 (13)	0.0577 (13)	0.0416 (11)	0.0026 (11)	−0.0016 (9)	−0.0006 (10)
C25	0.0476 (11)	0.0320 (10)	0.0362 (9)	0.0016 (8)	0.0030 (8)	0.0008 (8)
C26	0.0477 (11)	0.0290 (10)	0.0330 (9)	−0.0036 (8)	0.0065 (8)	−0.0011 (7)
C27	0.0530 (12)	0.0370 (11)	0.0397 (10)	−0.0076 (9)	0.0126 (9)	−0.0050 (8)
C28	0.0676 (16)	0.0712 (16)	0.0458 (12)	−0.0172 (12)	0.0170 (11)	0.0027 (11)
C29	0.096 (2)	0.093 (2)	0.0540 (14)	−0.0284 (17)	0.0323 (15)	−0.0033 (13)
C30	0.0540 (14)	0.0623 (15)	0.0593 (14)	−0.0010 (11)	0.0145 (11)	−0.0077 (11)
C31	0.0559 (16)	0.094 (2)	0.085 (2)	−0.0095 (14)	0.0192 (14)	−0.0171 (16)
C32	0.077 (2)	0.088 (2)	0.087 (2)	−0.0254 (16)	0.0448 (16)	−0.0155 (16)
C33	0.0403 (12)	0.0635 (15)	0.0676 (14)	0.0058 (10)	0.0102 (11)	0.0108 (11)
C34	0.0357 (13)	0.0710 (17)	0.105 (2)	0.0055 (11)	0.0063 (13)	0.0196 (15)
C35	0.0376 (13)	0.0639 (16)	0.103 (2)	0.0013 (11)	−0.0219 (13)	0.0163 (15)
C36	0.0480 (13)	0.0389 (11)	0.0662 (14)	−0.0002 (10)	−0.0210 (11)	0.0051 (10)
C37	0.0385 (11)	0.0320 (10)	0.0442 (10)	0.0014 (8)	−0.0092 (8)	0.0026 (8)
C38	0.0729 (18)	0.0573 (15)	0.0612 (15)	−0.0026 (13)	−0.0377 (13)	0.0075 (12)
C39	0.097 (2)	0.0507 (14)	0.0376 (11)	0.0020 (14)	−0.0225 (12)	0.0039 (10)
C40	0.0725 (15)	0.0303 (10)	0.0329 (10)	0.0008 (10)	−0.0026 (10)	0.0021 (8)
C41	0.0479 (11)	0.0273 (9)	0.0314 (9)	0.0001 (8)	−0.0054 (8)	0.0011 (7)
C42	0.098 (2)	0.0468 (13)	0.0333 (11)	0.0043 (13)	0.0142 (12)	0.0040 (9)
C43	0.0742 (17)	0.0529 (13)	0.0527 (13)	0.0043 (12)	0.0322 (12)	0.0046 (10)
C44	0.0454 (12)	0.0493 (12)	0.0451 (11)	0.0018 (9)	0.0100 (9)	0.0049 (9)

### Geometric parameters (Å, °)

**Table:**

Mn1—O1	2.1740 (14)	C16—H16	0.9300
Mn1—O3	2.1557 (15)	C17—C18	1.519 (2)
Mn1—O4^i^	2.1148 (14)	C18—C19	1.370 (3)
Mn1—O5	2.2358 (16)	C18—C20	1.428 (3)
Mn1—N3	2.2706 (16)	C19—C26	1.415 (2)
Mn1—N4	2.2914 (15)	C19—H19	0.9300
O1—C1	1.263 (2)	C20—C21	1.421 (3)
O2—C1	1.248 (2)	C20—C25	1.422 (3)
O3—C17	1.258 (2)	C21—C22	1.366 (3)
O4—C17	1.250 (2)	C21—H21	0.9300
O4—Mn1^i^	2.1148 (14)	C22—C23	1.400 (3)
O5—H5A	0.854 (17)	C22—H22	0.9300
O5—H5B	0.832 (16)	C23—C24	1.352 (3)
O6—H6A	0.89 (5)	C23—H23	0.9300
O6—H6B	0.874 (18)	C24—C25	1.419 (3)
N1—C10	1.321 (3)	C24—H24	0.9300
N1—C9	1.362 (3)	C26—C27	1.494 (3)
N2—C26	1.324 (2)	C27—C30	1.389 (3)
N2—C25	1.367 (2)	C27—C28	1.394 (3)
N3—C33	1.318 (3)	C28—C29	1.383 (3)
N3—C37	1.356 (2)	C28—H28	0.9300
N4—C44	1.321 (2)	C29—C32	1.371 (4)
N4—C41	1.360 (2)	C29—H29	0.9300
C1—C2	1.523 (3)	C30—C31	1.389 (3)
C2—C3	1.361 (3)	C30—H30	0.9300
C2—C4	1.440 (3)	C31—C32	1.379 (4)
C3—C10	1.423 (3)	C31—H31	0.9300
C3—H3	0.9300	C32—H32	0.9300
C4—C5	1.406 (3)	C33—C34	1.391 (3)
C4—C9	1.429 (3)	C33—H33	0.9300
C5—C6	1.370 (3)	C34—C35	1.355 (4)
C5—H5	0.9300	C34—H34	0.9300
C6—C7	1.407 (4)	C35—C36	1.394 (3)
C6—H6	0.9300	C35—H35	0.9300
C7—C8	1.346 (4)	C36—C37	1.412 (2)
C7—H7	0.9300	C36—C38	1.437 (3)
C8—C9	1.422 (3)	C37—C41	1.442 (3)
C8—H8	0.9300	C38—C39	1.336 (3)
C10—C11	1.479 (3)	C38—H38	0.9300
C11—C14	1.386 (3)	C39—C40	1.430 (3)
C11—C12	1.393 (3)	C39—H39	0.9300
C12—C13	1.385 (4)	C40—C42	1.397 (3)
C12—H12	0.9300	C40—C41	1.410 (2)
C13—C16	1.362 (4)	C42—C43	1.357 (3)
C13—H13	0.9300	C42—H42	0.9300
C14—C15	1.385 (3)	C43—C44	1.398 (3)
C14—H14	0.9300	C43—H43	0.9300
C15—C16	1.372 (4)	C44—H44	0.9300
C15—H15	0.9300		
			
O4^i^—Mn1—O3	93.06 (5)	C19—C18—C20	119.09 (16)
O4^i^—Mn1—O1	93.63 (6)	C19—C18—C17	117.88 (16)
O3—Mn1—O1	93.45 (5)	C20—C18—C17	123.01 (16)
O4^i^—Mn1—O5	83.35 (5)	C18—C19—C26	120.86 (17)
O3—Mn1—O5	173.29 (5)	C18—C19—H19	119.6
O1—Mn1—O5	92.44 (5)	C26—C19—H19	119.6
O4^i^—Mn1—N3	101.43 (6)	C21—C20—C25	118.27 (17)
O3—Mn1—N3	89.76 (5)	C21—C20—C18	125.33 (17)
O1—Mn1—N3	164.42 (6)	C25—C20—C18	116.37 (16)
O5—Mn1—N3	85.41 (6)	C22—C21—C20	120.49 (19)
O4^i^—Mn1—N4	173.08 (6)	C22—C21—H21	119.8
O3—Mn1—N4	91.24 (5)	C20—C21—H21	119.8
O1—Mn1—N4	91.51 (6)	C21—C22—C23	121.0 (2)
O5—Mn1—N4	91.81 (5)	C21—C22—H22	119.5
N3—Mn1—N4	73.16 (6)	C23—C22—H22	119.5
C1—O1—Mn1	125.43 (13)	C24—C23—C22	120.2 (2)
C17—O3—Mn1	131.91 (12)	C24—C23—H23	119.9
C17—O4—Mn1^i^	135.50 (12)	C22—C23—H23	119.9
Mn1—O5—H5A	97.9 (18)	C23—C24—C25	121.04 (19)
Mn1—O5—H5B	114.8 (18)	C23—C24—H24	119.5
H5A—O5—H5B	109 (2)	C25—C24—H24	119.5
H6A—O6—H6B	109 (4)	N2—C25—C24	117.91 (16)
C10—N1—C9	118.48 (18)	N2—C25—C20	123.08 (17)
C26—N2—C25	119.27 (15)	C24—C25—C20	119.01 (18)
C33—N3—C37	118.26 (17)	N2—C26—C19	121.24 (17)
C33—N3—Mn1	126.01 (14)	N2—C26—C27	117.69 (16)
C37—N3—Mn1	115.71 (12)	C19—C26—C27	121.07 (17)
C44—N4—C41	118.01 (16)	C30—C27—C28	118.04 (19)
C44—N4—Mn1	126.95 (13)	C30—C27—C26	121.57 (17)
C41—N4—Mn1	114.80 (12)	C28—C27—C26	120.35 (19)
O2—C1—O1	125.48 (19)	C29—C28—C27	120.7 (2)
O2—C1—C2	117.87 (18)	C29—C28—H28	119.7
O1—C1—C2	116.57 (19)	C27—C28—H28	119.7
C3—C2—C4	118.27 (18)	C32—C29—C28	120.8 (2)
C3—C2—C1	118.76 (18)	C32—C29—H29	119.6
C4—C2—C1	122.91 (19)	C28—C29—H29	119.6
C2—C3—C10	121.80 (19)	C27—C30—C31	120.8 (2)
C2—C3—H3	119.1	C27—C30—H30	119.6
C10—C3—H3	119.1	C31—C30—H30	119.6
C5—C4—C9	118.26 (19)	C32—C31—C30	120.3 (3)
C5—C4—C2	125.80 (19)	C32—C31—H31	119.8
C9—C4—C2	115.9 (2)	C30—C31—H31	119.8
C6—C5—C4	121.2 (2)	C29—C32—C31	119.4 (2)
C6—C5—H5	119.4	C29—C32—H32	120.3
C4—C5—H5	119.4	C31—C32—H32	120.3
C5—C6—C7	120.1 (3)	N3—C33—C34	123.1 (2)
C5—C6—H6	119.9	N3—C33—H33	118.5
C7—C6—H6	119.9	C34—C33—H33	118.5
C8—C7—C6	120.5 (2)	C35—C34—C33	118.9 (2)
C8—C7—H7	119.8	C35—C34—H34	120.6
C6—C7—H7	119.8	C33—C34—H34	120.6
C7—C8—C9	121.1 (2)	C34—C35—C36	120.7 (2)
C7—C8—H8	119.4	C34—C35—H35	119.7
C9—C8—H8	119.4	C36—C35—H35	119.7
N1—C9—C8	117.1 (2)	C35—C36—C37	116.6 (2)
N1—C9—C4	124.13 (19)	C35—C36—C38	124.8 (2)
C8—C9—C4	118.7 (2)	C37—C36—C38	118.7 (2)
N1—C10—C3	121.3 (2)	N3—C37—C36	122.51 (19)
N1—C10—C11	117.27 (18)	N3—C37—C41	118.05 (15)
C3—C10—C11	121.37 (19)	C36—C37—C41	119.44 (18)
C14—C11—C12	118.2 (2)	C39—C38—C36	121.7 (2)
C14—C11—C10	121.5 (2)	C39—C38—H38	119.1
C12—C11—C10	120.3 (2)	C36—C38—H38	119.1
C13—C12—C11	120.1 (3)	C38—C39—C40	121.4 (2)
C13—C12—H12	120.0	C38—C39—H39	119.3
C11—C12—H12	120.0	C40—C39—H39	119.3
C16—C13—C12	121.2 (3)	C42—C40—C41	117.73 (18)
C16—C13—H13	119.4	C42—C40—C39	123.3 (2)
C12—C13—H13	119.4	C41—C40—C39	119.0 (2)
C15—C14—C11	120.7 (3)	N4—C41—C40	122.17 (18)
C15—C14—H14	119.7	N4—C41—C37	118.04 (15)
C11—C14—H14	119.7	C40—C41—C37	119.79 (17)
C16—C15—C14	120.5 (3)	C43—C42—C40	119.65 (19)
C16—C15—H15	119.8	C43—C42—H42	120.2
C14—C15—H15	119.8	C40—C42—H42	120.2
C13—C16—C15	119.3 (3)	C42—C43—C44	119.3 (2)
C13—C16—H16	120.3	C42—C43—H43	120.4
C15—C16—H16	120.3	C44—C43—H43	120.4
O4—C17—O3	125.87 (17)	N4—C44—C43	123.18 (19)
O4—C17—C18	116.12 (17)	N4—C44—H44	118.4
O3—C17—C18	117.94 (17)	C43—C44—H44	118.4

Symmetry codes: (i) −*x*, −*y*, −*z*+2.

### Hydrogen-bond geometry (Å, °)

**Table:**

*D*—H···*A*	*D*—H	H···*A*	*D*···*A*	*D*—H···*A*
O5—H5A···O2	0.85 (2)	1.81 (2)	2.630 (2)	161 (3)
O5—H5B···N2^ii^	0.83 (2)	2.04 (2)	2.868 (2)	176.
O6—H6A···O1^iii^	0.89 (5)	2.30 (5)	3.175 (3)	167.
O6—H6B···N1^iv^	0.87 (2)	2.23 (2)	3.051 (3)	157.

Symmetry codes: (ii) −*x*, *y*+1/2, −*z*+3/2; (iii) *x*, −*y*+1/2, *z*−1/2; (iv) −*x*+1, *y*+1/2, −*z*+3/2.
